# Production of Myco-Nanomaterial Products from *Pleurotus ostreatus* (Agaricomycetes) Mushroom via Pyrolysis

**DOI:** 10.3390/pharmaceutics17050591

**Published:** 2025-04-30

**Authors:** Gréta Törős, Áron Béni, Andrea Kovács Balláné, Dávid Semsey, Aya Ferroudj, József Prokisch

**Affiliations:** 1 Institute of Animal Science, Biotechnology and Nature Conservation, Faculty of Agricultural and Food Sciences and Environmental Management, University of Debrecen, Böszörményi Street 138, 4032 Debrecen, Hungary; semi@gmail.com (D.S.); ferroudj.aya@agr.unideb.hu (A.F.); jprokisch@agr.unideb.hu (J.P.); 2 Doctoral School of Animal Husbandry, Faculty of Agricultural and Food Sciences and Environmental Management, University of Debrecen, Böszörményi Street 138, 4032 Debrecen, Hungary; 3 Institute of Agricultural Chemistry and Soil Science, Faculty of Agricultural and Food Sciences and Environmental Management, University of Debrecen, Böszörményi Street 138, 4032 Debrecen, Hungary; beniaron@agr.unideb.hu (Á.B.); kovacsa@agr.unideb.hu (A.K.B.); 4 Doctoral School of Nutrition and Food Science, University of Debrecen, Böszörményi Street 138, 4032 Debrecen, Hungary

**Keywords:** edible mushroom, fluorescence, carbon nanodots, thermal decomposition

## Abstract

**Background:** The study aimed to develop a sustainable method for producing myco-nanomaterials, particularly fluorescent carbon nanodots (CNDs), from freeze-dried *Pleurotus ostreatus* (Agaricomycetes) mushroom powder via pyrolysis. The goal was to investigate how pyrolysis conditions affect CND characteristics and their potential antimicrobial properties. Mushroom powder was pyrolyzed at temperatures ranging from 150 to 240 °C. The resulting products were analyzed for yield, molecular weight, fluorescence intensity, and estimated CND concentration in relation to the carbon-to-nitrogen (C/N) ratio. Antibacterial activity was tested against *Escherichia coli* and *Staphylococcus epidermidis*. Product yield decreased from 13.20% at 150 °C to 0.80% at 240 °C. Molecular weight peaked at 200 °C (623.20 kDa), while maximum fluorescence intensity (739.40 A.U.) was observed at 210 °C. A strong positive correlation (R^2^ = 0.72) was found between the C/N ratio and estimated CND concentration. Antimicrobial testing revealed notable inhibition of *E. coli,* associated with higher fluorescence intensity and CND content. Pyrolyzed *P. ostreatus* mushroom powder offers a promising, eco-friendly platform for producing luminescent, carbonaceous nanomaterials with antibacterial potential. These non-purified, myco-derived nanomaterials may contribute to green nanotechnology development and antimicrobial strategies.

## 1. Introduction

Nanotechnology is an interdisciplinary field that applies biotechnological methods to produce nanoparticles (NPs) with sizes typically below 10 nm. These nanoparticles are used for a variety of applications, including the treatment of diseases such as infections [1], cancer [2,3,4], and in several technological sectors, such as agriculture and food production [5]. Among the various types of nanoparticles, carbon nanodots (CNDs) have attracted significant attention due to their remarkable properties, including low toxicity, excellent water solubility, tunable fluorescence, high chemical stability, and the ease with which their surfaces can be functionalized [6,7]. These unique features make CNDs promising for bioimaging, drug delivery, environmental monitoring, and food safety applications [8]. Additionally, the ability to produce CNDs from renewable or waste-derived carbon sources makes them a sustainable and cost-effective alternative to other nanomaterials [9].

A key mechanism in the synthesis of CNDs is the Maillard reaction (MR), in which reducing sugars react with amino groups to form nitrogen-doped carbon structures that exhibit fluorescence [10]. In addition to MR, pyrolysis, a thermochemical process that decomposes organic matter at high temperatures without oxygen, is another common pathway for CND synthesis. Different variants of pyrolysis, such as fast, slow, catalytic, and flash pyrolysis, have been employed to process biomass, allowing for adjustments in thermal conditions and reaction times, all while maintaining an oxygen-limited environment [11,12,13]. Other significant methods for CND production include hydrothermal synthesis [14] and solvent-free carbonization [15], both of which promote the formation of carbon cores and surface functionalization, with tunable properties like size, crystallinity, and optical behavior depending on temperature, duration, and feedstock composition [14,15,16].

Myco-nanotechnology, a growing subfield that combines mycology with nanotechnology, leverages the biomass of edible and medicinal mushrooms to produce nanoparticles [17]. Mushrooms, such as *Pleurotus ostreatus,* are valuable for their nutritional and medicinal properties and potential in sustainable nanoparticle production [17,18]. The use of mushroom byproducts for nanomaterial synthesis offers an eco-friendly alternative to traditional synthetic routes, providing organic nanoparticles that are more digestible and biocompatible compared to inorganic counterparts [19]. These properties make mushroom-derived nanomaterials highly relevant for applications in human nutrition and medicine [20,21].

In biomedicine, one of the significant challenges is the growing problem of antibiotic resistance, which has made pathogens like *Escherichia coli* and *Staphylococcus aureus* increasingly challenging to treat with conventional antibiotics [22]. Recent studies have shown that CNDs possess antimicrobial properties, making them promising candidates in the fight against antibiotic-resistant bacteria [23]. However, the mechanisms by which CNDs exert these effects are still not fully understood, especially when derived from mushrooms through pyrolysis. Previous pyrolytic studies on organic material have failed to clearly define the mechanisms of action, especially regarding the formation of CNDs from mushroom biomass, due to the variability of thermal decomposition under different conditions [24]. Further research is necessary to elucidate these mechanisms and optimize synthesis parameters.

Kinetic analysis has been performed by Guo et al. (2015) [25] and Meng et al. (2024) [26], who investigated the practical usage of mushrooms in combination with another biomass source as a method for producing nitrogen-doped carbon catalysts. It has been evidenced that the mushroom is a promising candidate for the synthesis of NPs; however, final products can be affected by the carbon (C) and nitrogen (N) content. For example, higher nitrogen content resulted in nitrogen-doped carbon structures. The creation of catalytic surfaces by increased reactivity and porosity in CNDs can be affected by a higher C/N ratio, which results in increased reactivity [25,26].

*Pleurotus ostreatus* (oyster mushroom), rich in amino acids and sugars [27], serves as an excellent precursor for CND production through MR, offering a natural, biodegradable source for nanomaterial synthesis. Using *Pleurotus ostreatus* (oyster mushroom) as a precursor for CND production offers several advantages over other mushrooms and biosources. *P. ostreatus* is rich in amino acids, making it an ideal candidate for nitrogen-doped carbon structures that enhance the fluorescence and reactivity of the resulting nanodots.

Compared to other mushrooms, *P. ostreatus* provides a consistent and abundant supply of these essential precursors [27], making it a more reliable source for large-scale nanoparticle synthesis.

This research aims to develop a cost-effective and environmentally sustainable method for producing carbon-rich nanomaterial fractions from *Pleurotus ostreatus* mushroom powder via pyrolysis. Specifically, we seek to (i) enhance the biological activity of the water-soluble fractions derived from freeze-dried, pyrolytic processed *P. ostreatus* (ii), evaluate the influence of the carbon-to-nitrogen (C/N) ratio on the concentration of carbon nanodots (CNDs), and (iii) investigate the surface chemistry and size distribution of CND-enriched organic mushroom products (OMPs). Additionally, we will assess the antimicrobial activity of the pyrolyzed samples against *Escherichia coli* and *Staphylococcus epidermidis*. Ultimately, this work aims to identify bioactive, myco-derived nanomaterials with potential applications in preventing and treating infectious diseases, contributing to global efforts to combat antimicrobial resistance.

## 2. Materials and Methods

### 2.1. Synthesis of Oyster Mushroom-Based Carbon Nanodots and Experimental Design

**Figure 1:**
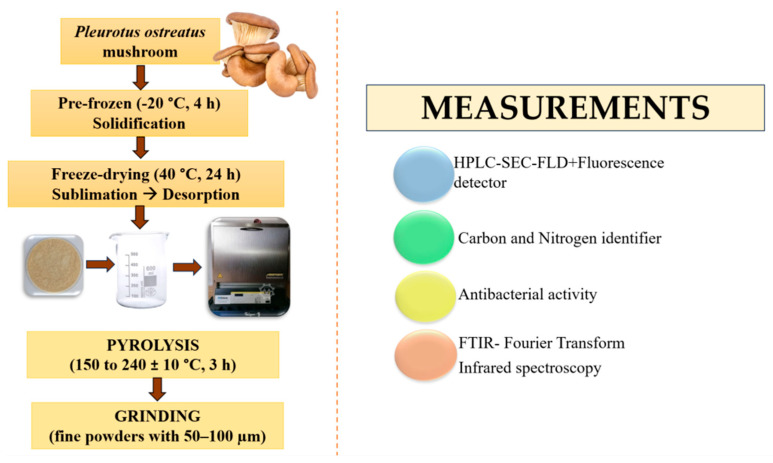
Starter material (freeze-dried *P. ostreatus* mushroom), processing method (pyrolyzed freeze-dried mushroom powder), and summary of analytical measurements performed.

Fresh oyster mushrooms (OM) were sourced from a local supermarket in Debrecen (Hungary). The manufacturing process of the samples investigated was performed at the Nanofood Laboratory, Institute of Animal Science, Biotechnology, and Nature Conservation. Figure 1 provides a photographic illustration of the applied research protocol and the main steps of production.

After thorough washing, the *Pleurotus ostreatus* mushrooms were manually cut into quarters and pre-frozen at −20 °C for 4 h to preserve structural integrity and optimize their condition for further processing. Following pre-freezing, the samples underwent freeze-drying, conducted by Bionanoferm Ltd. (Debrecen, Hungary). During this process, the chamber pressure was reduced to <0.1 mbar, and the frozen water was removed via sublimation. Initially, the samples were held at low temperatures to allow sublimation, followed by a secondary drying phase at 40 °C for 24 h to eliminate residual moisture. After lyophilization, the samples were ground under standardized conditions using a Bosch coffee grinding machine for 150 g/min, yielding a fine oyster mushroom powder (OMP) with a particle size distribution of 50–100 µm at room temperature (23–25 °C). Subsequently, the OMP samples were subjected to pyrolysis to synthesize carbon nanodots (OMP-CNDs). The pyrolysis was carried out in a controlled environment using an ash furnace (Nabertherm GmbH, Lilienthal, Germany). Approximately 10 g of OMP was placed in a 100 mL glass beaker, which was covered with a glass Petri dish to maintain a semi-closed system. The pyrolysis process was conducted over 3 h at each set temperature, starting from 150 °C and increasing in 10 °C increments up to 240 °C. The control group (unprocessed freeze-dried samples) was prepared only to characterize the final product (microbiological and Fourier Transform Infrared Spectroscopy (FTIR) investigations).

The following sample preparations were applied to analyze the characteristics of potential nanomedicines:

(1) Molecular weight (kDa), fluorescence intensity (A.u.), and **OMP**-based **CND** (**OMP-CND**) concentration (mg/kg) were determined using high-performance liquid chromatography. For this analysis, pyrolyzed dry powders (processed at 150 °C to 240 °C) were diluted 100-fold in distilled water, vortexed (15 s), and filtered through a 0.45 µm hydrophilic PTFE syringe filter (Labex Ltd., New Delhi, India).
(2) To evaluate the carbon and nitrogen (C/N) ratio and conduct UV–visible spectrophotometry, pyrolyzed dry powders were diluted ten-fold in distilled water, vortexed for 15 s, and exposed to ultrasonic cleaner (Olympus, Tokyo, Japan) treatment (30 kHz) for 20 min. The processed samples were filtered twice with filter paper (100 µm dairy filter paper). The water-soluble fractions were pre-frozen and freeze-dried at 40 °C for approximately 24 ± 2 h. These final samples (oyster mushroom powder with enhanced carbon nanodots content from the freeze-dried water-soluble fraction: OMP-CND-FL) were analyzed for their C/N ratio and fluorescence spectra.
(3) To investigate the final product, Fourier Transform Infrared Spectroscopy (FTIR) and antibacterial activity assays were used, including evaluating antimicrobial effects against *Escherichia coli* and *Staphylococcus epidermidis*. FTIR investigations were performed on pyrolyzed (with the highest fluorescence intensity and CND concentration) and non-pyrolyzed freeze-dried OMPs samples.

### 2.2. Yield of Freeze-Dried Water-Soluble Myco-Nanomaterial Products

The yield of OMP-CND-FL from the initial material (freeze-dried oyster mushroom powder) was calculated as described in the following equation:

$$Yield\;\left(\%\right)=\frac{\mathrm{Mass}\;\mathrm{of}\;\mathrm{water}\;-\mathrm{soluble}\;\mathrm{CND}\;fraction\;produced\;by\;pyrolisis}{Initial\;mass\;of\;freeze-dried\;mushroom\;powder\;before\;pyrolisis}\times 100\;$$

### 2.3. Characterization of Mushroom-Based Carbon Nanodots

High-performance liquid chromatography (HPLC) analysis was conducted at the Institute of Agricultural Chemistry and Soil Science lab at the University of Debrecen, and spectrofluorimetric measurements were performed at the Faculty of Nature Science and Technology.

HPLC analysis of oyster mushroom powder-based carbon nanodots (OMP-CNDs) was conducted following the methodology outlined by Nguyen et al. (2024), which examines the potential formation of CNDs during the thermal processing of food products and the associated heat-induced reactions [28]. Furthermore, after low-temperature, long-duration cooking (at 90 °C for 4 h), our group successfully tested mushroom-derived carbon nanodots beforehand [29].

The High-Performance Liquid Chromatography–Size Exclusion Chromatography–Fluorescence Detection (HPLC-SEC-FLD) measurements utilized size-exclusion chromatography to determine the molecular size and mass of the OMP components. 

Sample separation was carried out using an Agilent AdvanceBio SEC column (300 Å; 4.6 × 300 mm × 2.7 μm) with an isocratic elution system. The system incorporated a Shimadzu RF-20A fluorescence detector (Kyoto, Japan) linked to an HPLC unit (ECOM, ECS05, Chrastany u Prahy, Czech Republic). The circumstances of the measurements and details are shown in Table 1.

The system was calibrated using two peptide standard mixtures to determine the molecular weight, as summarized in Table 2. These standards facilitated precise calibration and molecular weight identification of CNDs in the tested samples [28]. A calibration curve with known concentrations was then developed to determine the final CND concentration in the OMP samples.

Water-extracted OMP with CND content from *P. ostreatus* mushroom powder (OMP-CNDs) were analyzed using an FP-8500 fluorescence spectrophotometer (Jasco Co., Ltd., Oklahoma City, OK, USA). The spectrofluorometric measurements were conducted at an excitation wavelength of 370 nm, following established methodologies [28].

Furthermore, our HPLC method allows for estimating particle size distribution (PSD) and polydispersity (PD) by analyzing the shape and breadth of the fluorescence peaks, as supported by Gromotka et al. (2022) [30].

$$PD=\frac{Mw}{Mn}$$

where Mw = Weight-average molecular weight, Mn = Number-average molecular weight.

### 2.4. Measurement of Carbon and Nitrogen (C/N) Content

The Dumas method for determining nitrogen content involves the complete combustion of a sample (approximately 0.1 g) at around 1150 °C in an oxygen-rich environment. During this process, the sample is oxidized, producing combustion gases such as O_2_, CO_2_, H_2_O, N_2_, and nitrogen oxides. These gases are collected and passed through a series of traps to remove selectively all components except nitrogen and nitrogen oxides. The remaining gas mixture is then directed through a catalytic post-combustion zone and into a reduction zone, where hot tungsten reduces nitrogen oxides to molecular nitrogen and removes excess oxygen [31].

Using CO_2_ as the carrier gas, the resulting mixture is transferred through a two-stage drying system and an electronic flow controller before reaching a thermal conductivity detector (TCD; Agilent Technologies, Santa Clara, CA, USA). The nitrogen content is quantified based on the thermal conductivity of N_2_, while CO_2_ is monitored by Near-Infrared Spectroscopy (NIR; Bruker Optics, Billerica, MA, USA). The final nitrogen concentration is calculated using the detector signals and the sample’s weight [32].

### 2.5. Investigations of the Antibacterial Activity of the Final Product

Microbiological research was conducted at the Nanofood Laboratory, Institute of Animal Science, at the University of Debrecen. This utilized microbiological methods to assess the ability of pyrolyzed oyster mushroom powders to inhibit the growth of harmful bacteria. To prepare the starter cultures, sterile nutrient broths (MRS, BioLab Zrt., Budapest, Hungary) were inoculated with rehydrated bacterial colonies of *Escherichia coli* (B.02357) and *Staphylococcus epidermidis* (B.02055) sourced from the reference collection of the Hungarian University of Agriculture and Life Sciences, which were collected from solid MRSA agar using sterile cell scrapers. The cultures were vortexed and then incubated overnight at 37 °C, and starter cultures with OD values over 4 were used to perform the test.

Microbiological experiments were performed using one specific group to analyze the functional properties of the pyrolyzed oyster mushroom powders (OMPs). A particular sample was chosen according to the experimental results of the fluorescence intensity (A.u) and final concentrations (mg/kg) of CND, where the highest results were achieved. The final freeze-dried, pyrolyzed samples (OMP-CNDs) were used against two bacteria and compared with the control sample without added supplementation.

OMP-CNDs were applied at one specific concentration, 1 (*w*/*v*%). Following our previously described methodology, we performed growth inhibition, which was calculated using a negative control. We selected 1 (*w*/*v*%) based on the results of poultry experiments, in which we had already performed microbiological experiments with OMPs produced by different technologies [28].

### 2.6. Results for Fourier Transform Infrared Spectroscopy Analysis

Fourier Transform Infrared Spectroscopy (FTIR) was performed at the University of Debrecen (Faculty of Agricultural and Food Sciences and Environmental Management, Institute of Water and Environmental Management) to investigate the functional groups present on the surface of the CND-rich OMPs. The analysis was conducted using a Jasco 615-type spectrophotometer (or equivalent) in the 400–4000 cm^−1^ range with a resolution of 2 cm^−1^. Fine powder samples were used (freeze-dried + untreated and freeze-dried + pyrolyzed (210 °C, 3 h) oyster mushroom powder). The spectra were recorded over four scans for each sample, and the background spectra were collected under identical conditions. The characteristic peaks corresponding to various functional groups, such as hydroxyl (–OH), carbonyl (C=O), and amine (–NH_2_) groups, were identified based on the reference FTIR data of known compounds according to Baudot et al. (2010) [33]. The peaks were assigned according to standard FTIR spectral databases, and the intensity of specific peaks was correlated with the properties of the CNDs, such as their fluorescence intensity and stability.

### 2.7. Statistical Analysis

Statistical analysis was carried out using SPSS software (version 25.0). After testing extreme values, we then test for normality (Shapiro–Wilk test). An ANOVA test was applied, followed by Tukey’s post hoc test, to normally distributed datasets for data comparison, while the Kruskal–Wallis test, followed by Dunn’s post hoc test, was used for non-normally distributed data. Differences were considered statistically significant at *p* < 0.05. Each sample originated from one specific batch, which was analyzed in triplicate, and all charts were created using Microsoft Excel 365 Edition. Furthermore, the relationship between the C/N ratio and OMP-CND concentration was also assessed by correlation analysis.

## 3. Results

### 3.1. Yield of Water-Soluble OMP-CNDs at Different Temperatures

Understanding the yield of pyrolyzed *P. ostreatus* mushroom powder (OMP-CND-FL) from a given amount of freeze-dried raw material is essential. In this study, each treatment started with 10 g of freeze-dried mushroom powder, which required a minimum amount of 100 g of fresh mushroom. The first step involved determining the mass reduction (%) of the pyrolyzed *P. ostreatus* powder (OMP-CND). Subsequently, a 10× aqueous solution was prepared, following the described protocol. The yield of OMP-CND-FL was calculated as the percentage of the aqueous fraction relative to the initial sample weight before pyrolysis, as shown in Table 3.

The results showed a clear trend: the yield (%) generally decreased as the temperature increased. The highest yield was measured at the lowest temperature tested (150 °C), while the lowest yield was seen at the highest temperature (240 °C). Statistical analysis (*p* = 0.028) confirmed significant differences between the treatments. The yields at 230 °C and 240 °C were much lower than those at 150 °C, 170 °C, and 220 °C. In addition, the yield at 190 °C was also significantly lower than that at 150 °C (*p* = 0.018) and 220 °C (*p* = 0.042).

### 3.2. Changes in Molecular Weight of CND Affected by Pyrolitic Heating Processes

Table 4 presents the HPLC analysis results, including integrated molecular weight estimations derived from peak positions and calibration data. Statistical analysis revealed significant differences in the molecular weights (kDa) of OMP-CNDs samples treated at different temperatures (*p* = 0.034). According to the results, pyrolysis at higher temperatures (180, 190, and 200 °C) increased the molecular weight compared to heat treatments at 150 and 160 °C. In contrast, treatments at 230 °C (*p* = 0.028) and 240 °C (*p* = 0.031) significantly decreased the molecular weight relative to the sample treated at 190 °C.

### 3.3. Changes in Fluorescence Intensity and CND Concentration Affected by Pyrolitic Heating Processes

**Figure 2:**
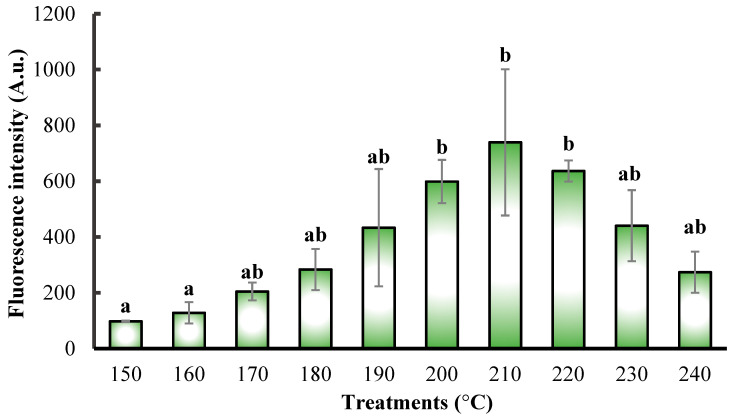
Fluorescence intensity (A.u.) of OMP-CND samples pyrolyzed at different temperatures (150–240 °C). Significant differences are indicated by different letters (a,b).

Our analysis examined the summarized fluorescence intensity of higher and lower peak heights split by dimerization, and we found significant differences between different treatments (*p* = 0.046). By comparing the fluorescence intensity, we found that the fluorescence intensity (A.u.) of the samples treated at 200 °C, 210 °C, and 220 °C was significantly higher than those treated at 150 °C and 160 °C. The lowest average fluorescence intensity was observed in the samples treated at 150 °C with a value of 97.99 ± 1.84 A.u., and the highest intensity was recorded at 210 °C with 739.40 ± 184.47 A.u., as shown in Figure 2.

**Figure 3:**
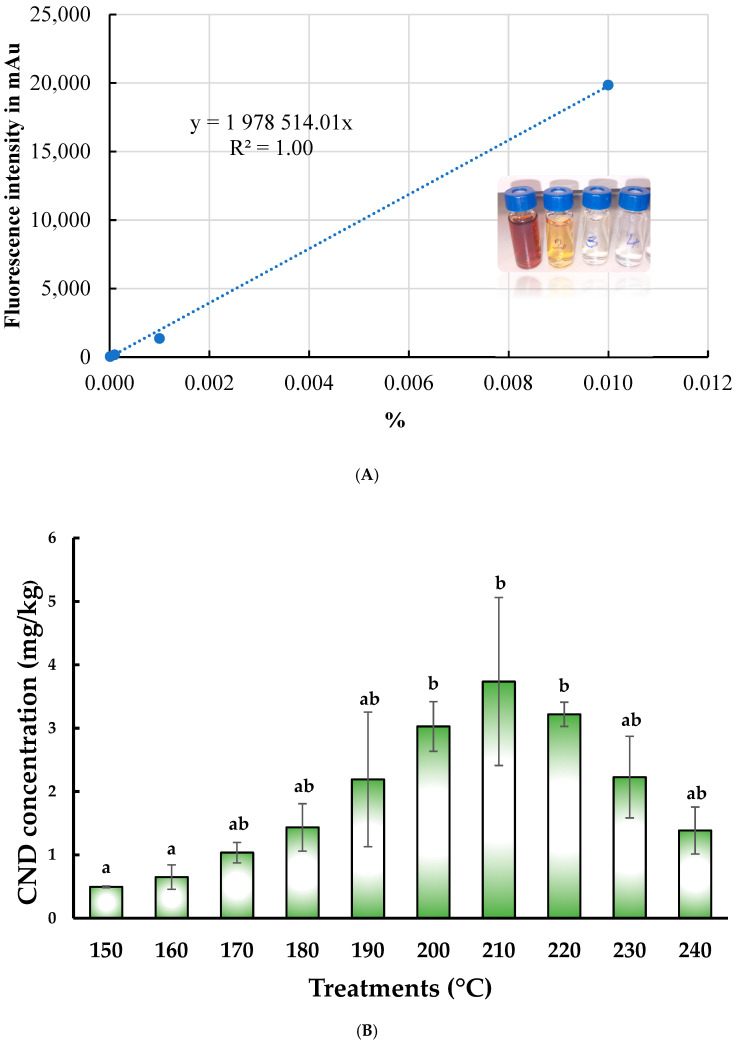
(**A**) Calibration curve used for the quantification of carbon nanodot concentration; (**B**) CND concentration (mg/kg) in pyrolyzed OMP-CND samples treated at various temperatures. Significant differences are indicated by different letters (a,b).

As the first step in determining the final concentration of CND (mg/kg), we prepared the calibration series (a single-point measurement, illustrated in Figure 3B), measured the fluorescence intensity corresponding to the given points, and then constructed the calibration curve (Figure 3A).

After summing the fluorescence intensities of the two peaks for the samples to be analyzed, we calculated the carbon nanoparticle concentration (mg/kg) using the calibration curve, as shown in Figure 3A During pyrolysis, the carbon nanoparticle concentration varied significantly among the groups treated at different temperatures (*p* = 0.046). 

**Figure 4:**
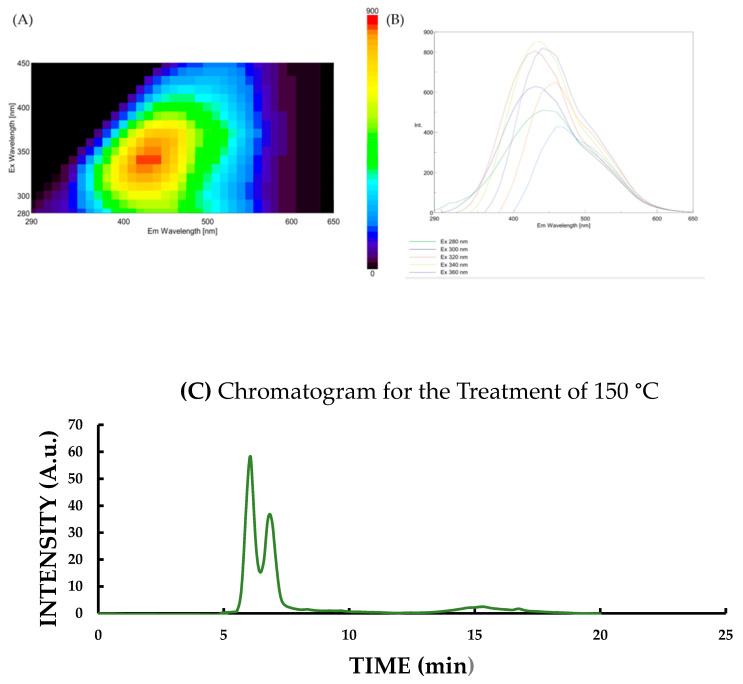

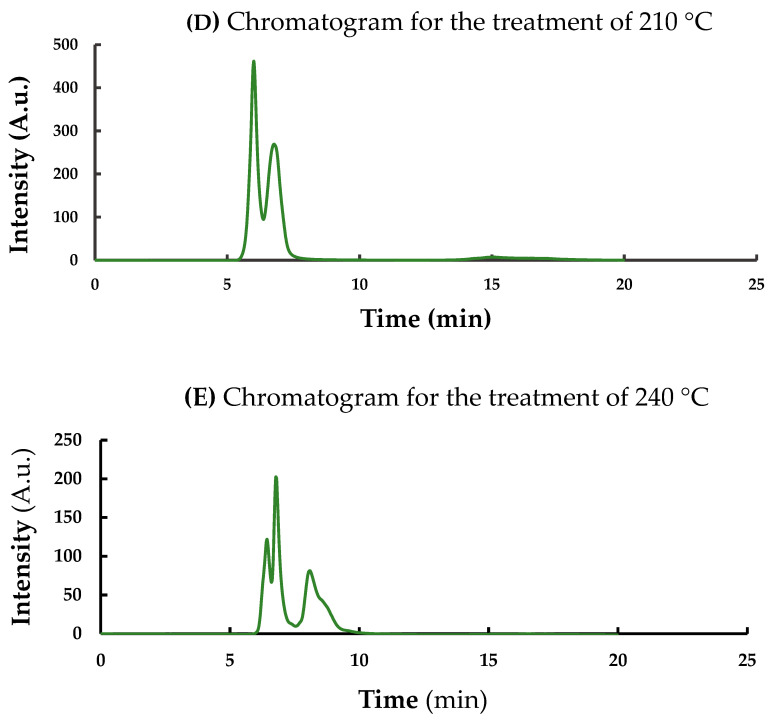
Representative fluorescence data of OMP-CNDs measured using a Jasco FP-8500 fluorescence spectrophotometer. (**A**) 3D fluorescence spectra of selected samples; (**B**) Wavelength-resolved fluorescence profiles showing intensity variation. Fluorescence chromatograms of OMP-CNDs measured by HPLC with fluorescence detection: (**C**) 150 °C, (**D**) 210 °C, and (**E**) 240 °C samples. Distinct peaks are observed near 5 min.

The same tendency was calculated for the final concentration of CND, as measured for fluorescence intensity. The lowest concentration was measured at 150 °C (Figure 4C), while the highest was obtained at 210 °C (Figure 4D).

After analyzing the data, which did not follow a normal distribution, we found a significant difference between the groups treated at 150 °C (0.50 ± 0.01 mg/kg) and 160 °C (0.65 ± 0.19 mg/kg) compared to those treated at 200 °C (3.03 ± 0.39 mg/kg), 210 °C (3.74 ± 1.33 mg/kg), and 220 °C (3.22 ± 0.19 mg/kg), with higher levels resulting from temperatures over 200 °C. 

Figure 4A,B present the results obtained after fluorescence measurements: 3D fluorescence spectra (Figure 4A) and the corresponding emission/excitation wavelength spectra (Figure 4B). Additionally, Figure 4C, Figure 4D and Figure 4E display chromatograms recorded at the lowest tested temperature (150 °C), the most effective temperature yielding the highest fluorescence intensity (210 °C), and the highest tested temperature (240 °C), respectively, clearly illustrate the temperature-dependent changes in fluorescence and chromatographic profiles of the non-purified OMPs with carbon nanoparticle content.

### 3.4. Evaluation of Size Distributions After Calculation of Polydispersity Index

**Figure 5:**
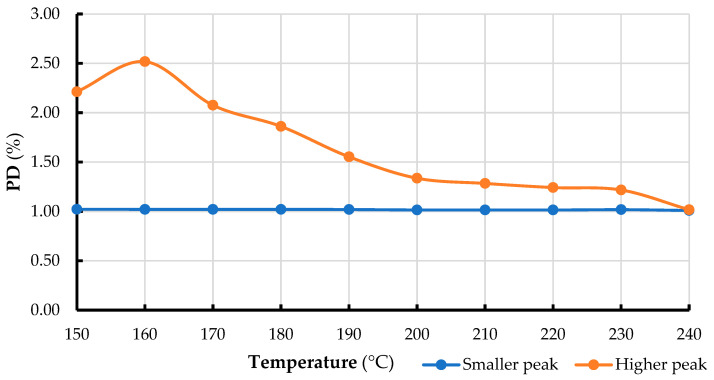
Polydispersity analysis based on peak broadness from HPLC-fluorescence profiles of OMP-CNDs treated at temperatures from 150 °C to 240 °C (10 °C increments). Both major and minor peaks were evaluated for polydispersity.

The results for the polydispersity (PD %), based on the shape and breadth of the elution peaks are shown in Figure 5. According to the HPLC analysis, the carbon nanoparticles are below 10 nm in size. A clear temperature-dependent trend emerged after calculating the polydispersity index (PD, dimensionless; see the table below). In summary, as the temperature decreases, the PD values for the prominent peaks increase notably, while the PD values for the small peaks remain relatively stable, with only minor fluctuations. Specifically, as the temperature drops from 250 °C to 150 °C, the PD of the prominent peaks increases significantly from 0.87 to 2.21%, indicating greater polydispersity at lower temperatures. In contrast, the PD of the minor peaks remains nearly constant, ranging narrowly between 1.01 and 1.03%.

### 3.5. Effect of Carbon and Carbon/Nitrogen Ratio on the Fluorescence Properties of Oyster Mushroom Powder After Pyrolysis

**Figure 6:**
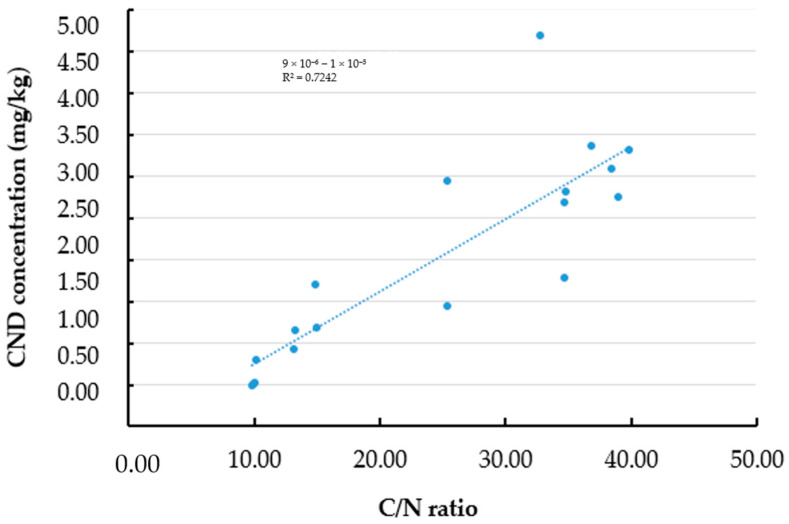
Relationship between the concentration of carbon nanodots (mg/kg) and the carbon-to-nitrogen (C/N) ratio (%) in the water-soluble fraction of pyrolyzed OMP-CND samples (treated at 150–240 °C).

The Pearson correlation analysis and scatter plot showed a positive correlation between the C/N ratio (%) and the CND concentration (*w*/*w*%) (R^2^ = 0.7242), which indicates that the linear relationship with the C/N ratio can explain 72.4% of the variance in CND concentration (*p* < 0.001), as shown in Figure 6.

### 3.6. Antibacterial Activity of P. ostreatus-Based CNDs

Freeze-dried, pyrolyzed (210 °C, 3 h) oyster mushroom-based products from the complex matrix of the water-soluble fraction exhibited notable antimicrobial activities against each tested harmful bacteria (*E. coli* and *S. epidermidis*), as summarized in Table 5. The antimicrobial activity was assessed to evaluate the effectiveness of the OMP-based carbon nanoparticle (CND) solutions (with a final volume of 10 mL) at different concentrations (1 *w*/*v*%).

Freeze-dried, pyrolyzed oyster mushroom-based products (from the complex matrix of the water-soluble matrix) exhibited potential antimicrobial activities against harmful bacteria (*E. coli*) (Table 5). During the antimicrobial activity tests for *S. epidermidis* and pour plate methods for *E. coli,* inhibition was illustrated as a million bacteria (CFU/g) mean ± SD for each microorganism by taking three replicates. After conducting an independent samples Mann–Whitney U Test on data that did not follow a normal distribution, it was determined that the tested myco-nanomaterial products exhibited a statistically significant inhibitory effect on the growth of *E. coli*. Specifically, the bacterial count in the treated samples was reduced compared to the control sample that lacked the test supplements (*p* = 0.029). Conversely, no significant inhibitory effect was observed against *S. epidermidis* (*p* > 0.05).

### 3.7. Results of FTIR Analysis

**Figure 7:**
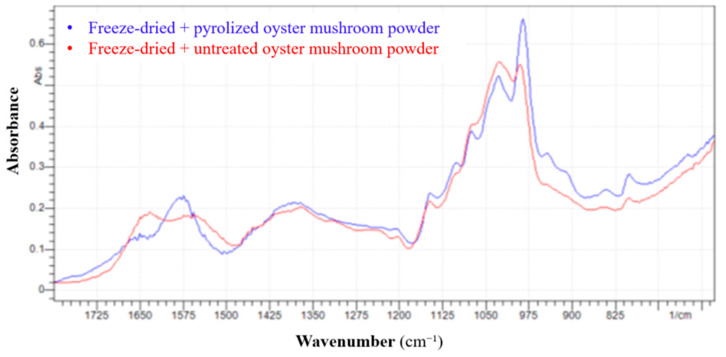
FTIR absorption spectra of freeze-dried untreated and pyrolyzed (210 °C, 3 h) *P. ostreatus* mushroom powder, highlighting changes in functional groups upon pyrolysis.

Figure 7 shows the results of FTIR experiments for freeze-dried + untreated and freeze-dried + pyrolyzed (210 °C, 3 h) oyster mushroom powder. The pyrolyzed sample shows higher absorbance intensity in several regions, particularly around 1050–950 cm^−1^, suggesting increased concentration or structural exposure of specific functional groups after thermal treatment. The untreated spectrum has broader peaks, indicating more complex overlapping signals due to intact biomolecules (e.g., polysaccharides, proteins).

Several notable changes were observed upon conducting a region-by-region comparison (1800–600 cm^−1^). For instance, in the ~1740–1600 cm^−1^ range, the broadband becomes sharper after pyrolysis, suggesting the decomposition or rearrangement of carbonyl-containing groups, such as carboxylic acids, esters, or amides. Furthermore, around ~1550–1500 cm^−1^, the pyrolysis process leads to sharpening and shifting peaks, pointing to modifications in protein structures or formation of aromatic compounds. A slight decrease in intensity is observed in the ~1400–1300 cm^−1^ range, reflecting the thermal degradation of aliphatic chains, including CH_2_ and CH_3_ groups. A sharp intensity increase in the ~1150–1000 cm^1^ range, particularly around 1050–975 cm^1^, signals carbohydrate dehydration and the formation of ether or alcohol structures. Finally, in the ~900–700 cm^−1^ range, the increased peak intensity suggests a rise in aromatic content due to partial carbonization during pyrolysis

## 4. Discussion

Myco-nanotechnology, which involves the synthesis of nanoparticles from edible mushrooms, is a promising technology that has attracted many studies [34,35]. The present study demonstrates the rapid and sustainable preparation of *Pleurotus ostreatus* (oyster mushroom) powder with considerable carbon nanodots (CNDs) via controlled pyrolysis. The effects of pyrolysis temperature on CND yield, size distribution, physicochemical characteristics, and antibacterial activity were systematically investigated in oyster mushroom powders.

Our findings confirmed that pyrolysis temperature significantly influenced the yield and characteristics of the resulting CNDs. Maximum recovery of CNDs (% dry mass) was achieved at 150 °C, suggesting limited degradation of thermolabile compounds at lower temperatures. In contrast, yields declined sharply above 230 °C, likely due to extensive thermal decomposition and the formation of volatile byproducts [36,37].

Molecular weight and pyrolysis temperature followed a non-linear relationship. Temperatures between 180–220 °C resulted in high molecular weight values (e.g., 614.60 kDa at 180 °C and 608.50 kDa at 220 °C), indicating a favorable balance between polymerization and thermal stability [38,39]. However, temperatures beyond 230 °C caused degradation of macromolecules, possibly via radical polymerization, cross-linking, and bond cleavage mechanisms. These changes are consistent with the behavior of bio-based materials containing β-glucans, proteins, and amino acids [40].

Dimerization of CNDs, observed via structural analysis (Figure 4C,D), suggests that nanoparticle aggregation may contribute to shifts in electronic transitions, ultimately affecting fluorescence and optical properties [41,42]. This structural change is temperature-dependent, as moderate thermal treatment promotes dimer formation, while excessive heating causes over-carbonization and reduced fluorescence [43,44].

Fluorescence intensity peaked between 200 °C and 220 °C, with a maximum at 210 °C (739.40 ± 184.47 A.u). Beyond this range, intensity declined, likely due to loss of structural integrity or increased non-radiative decay pathways [45,46,47]. In line with this, CND concentrations were highest at 200–220 °C, especially at 210 °C (3.74 mg/kg), following a 3-h pyrolysis in a closed system. Molecular weight, fluorescence intensity, and CND concentration collectively suggest that this temperature range is optimal for producing high-quality OMP-CNDs.

HPLC results showed that the carbon nanoparticles were smaller than 10 nm. As the pyrolysis temperature decreased from 240 °C to 150 °C, the main peaks’ polydispersity (PD) increased significantly (from 0.87 to 2.21%), indicating that particle size distribution became more varied. In contrast, the PDI of smaller peaks remained nearly constant (1.01–1.03%). This suggests that lower temperatures lead to greater polydispersity, highlighting the need for precise temperature control to produce uniform carbon nanoparticles.

We also identified a strong and statistically significant correlation between the carbon-to-nitrogen (C/N) ratio and CND concentration (*p* < 0.001). Approximately 72.4% of the variance in CND yield could be explained by changes in the C/N ratio, showing the importance of nitrogen content, which may enhance nanoparticle formation through mechanisms such as surface passivation or heteroatom doping [45,48,49]. These findings suggest that controlling elemental composition can support the synthesis of nanoparticles with valuable properties.

Our findings for antibacterial activity suggest that the tested myco-nanomaterial source effectively suppressed the proliferation of *E. coli*, demonstrating its potential as a novel and powerful antibacterial agent. The bacterial count (*E. coli*) in the treated samples was reduced to 605 ± 7.1 million CFU/g, representing a significant decrease compared to the control sample (1075 ± 16.1 million CFU/g) (*p* = 0.029). This result highlights the promising use of mushroom-derived carbon nanodots as an effective antimicrobial solution, particularly in the context of *E. coli* infections, which are a significant concern in clinical and food safety settings. While its activity next to selected concentrations against *S. epidermidis* remains inconclusive. Gram-negative bacteria, such as *E. coli*, were more susceptible to the mushroom-derived carbon nanodots than Gram-positive bacteria, likely due to the structural differences in their cell walls [50].

FTIR analysis at 210 °C revealed chemical transformations typical of early-stage pyrolysis. Simplified spectral features in the fingerprint region (1500–600 cm^1^), sharper bands at 1050–950 cm^1^, and increased carbon-rich signals support the formation of carbonaceous structures and dehydration of saccharides. These observations are consistent with the partial degradation of proteins and polysaccharides and the onset of Maillard-type reactions, contributing to nanoparticle formation.

Our study provides insight into the connection between the carbon-to-nitrogen ratio and the formation of carbon nanodots in freeze-dried oyster mushroom powder subjected to pyrolytic processing. Our findings indicate that suppressing harmful *E. coli* growth could offer a promising approach to reducing antibiotic reliance and combating antibiotic resistance.

Overall, 210 °C is most advantageous for optimal pyrolysis, as supported by multiple measured parameters, especially fluorescence intensity (739.40 ± 184.47 A.u), molecular weight (618.40 ± 1.80 kDa), and concentration (3.74 mg/kg).

Our findings underscore the promising potential of mushroom-derived carbon nanodots (CNDs); however, several critical steps remain before their practical application can be advanced. A significant challenge is the lack of purification, as the current material may contain residual organic compounds, salts, or pyrolysis by-products that could affect biological activity and compatibility with downstream uses. Future studies should involve a broader spectrum of Gram-positive and Gram-negative pathogens, incorporate standardized minimum inhibitory concentration (MIC) assays, and perform time-kill studies under controlled experimental conditions to better evaluate their antimicrobial efficacy.

## 5. Conclusions

There are little data about mushroom-based carbon nanodots, which are primarily based on laboratory studies [51,52]. The most frequent studies are on the green synthesis of silver nanoparticles from edible mushrooms [53,54]. Our research highlights the potential of oyster mushrooms (*Pleurotus ostreatus* L.), which contain quality reactants for the Maillard reaction, to play a pivotal role in sustainable nanomaterial synthesis, and offer potential biomedicinal applications.

Our study demonstrates the feasibility of synthesizing bioactive carbon nanodots (CNDs) from freeze-dried *Pleurotus ostreatus* mushroom powder (OMP) via pyrolysis under controlled conditions. The synthesized method in this study was rapid and safe for the environment, with several economic benefits. Our findings show the critical role of pyrolysis temperature in yield, molecular weight, and fluorescence properties of oyster mushroom-based CNDs, so the technology was successfully developed. Furthermore, the final product proved its effectiveness against *Escherichia coli*, and low-temperature heating is sufficient to alter the chemical fingerprint of biopolymers, providing a valuable strategy for tailoring natural materials in applications such as carbon nanomaterial synthesis.

Future investigations should focus on optimizing synthesis parameters and exploring the functional applications of different mushroom-based CNDs in practical settings, emphasizing precise mushroom-based CND manufacturing. For instance, a deeper investigation of the gut microbiota should be conducted. Clear carbon nanodots should also be produced from oyster mushroom and tested, especially against pathogens, to develop highly effective food or feed supplements to fight antibiotic resistance and further prove their effectiveness.

## Figures and Tables

**Table 1:** Description of the HPLC settings and applied conditions.

Parameter	Description
Mobile Phase	Acetonitrile: Water (20:80, *v*/*v*), unbuffered
Flow Rate	0.7 mL/min
Injection Volume	5 μL
Excitation Wavelength	370 nm
Emission Wavelength	460 nm
Scan Speed	Medium (600 nm/min)
Integration Time	1 s
Emission Scanning	Not performed (fixed excitation/emission)
Column Temperature	30 °C
Detector Temperature	Ambient (~25 °C)
Sample Preparation	100× dilution in distilled water, 0.45 μm hydrophilic PTFE filter (Labex Ltd.)
Mobile Phase pH	~6.0 (unbuffered)
Ionic Strength	Low (no salts or buffers added)

**Table 2:** Calibration and carbon nanodot (CND) determination.

Parameter	Description
Calibration Purpose	Molecular weight identification and quantification of CNDs
Standard Mix 1	Bio-Rad Gel Filtration Standard (MW range: 1350–670,000 Da)
Components (Standard 1)	Thyroglobulin, γ-globulin, ovalbumin, myoglobin, vitamin B12
Standard Mix 2	Merck Peptide Standards
Components (Standard 2)	Gly-Tyr (238.2 Da), Val-Tyr-Val (379.5 Da), Met-enkephalin (573.7 Da), Leu-enkephalin (555.6 Da), Angiotensin II (1046.2 Da)
CND Reference Standard	Synthesized from glycine and dextrose (Nguyen et al., 2024 [28])
Calibration Series	4-point series with concentrations: 10, 100, 1000, and 10,000 g/mL
Solvent for Calibration	Distilled water
Output	Calibration curve to quantify CNDs in OMP (mushroom) samples

**Table 3:** Yield (%) of pyrolytically processed oyster mushroom-based carbon nanodot (OMP-CND) samples treated at temperatures ranging from 150 °C to 240 °C.

Under 200 °C		Above 200 °C	
Temperature (°C)	Yield (%) ± SD	Temperature (°C)	Yield (%) ± SD
150	13.20 ± 0.41 ^b^	200	6.80 ± 0.26 ^ab^
160	7.00 ± 0.21 ^ab^	210	7.00 ± 0.22 ^ab^
170	7.60 ± 0.22 ^b^	220	8.00 ± 0.27 ^b^
180	6.60 ± 0.24 ^ab^	230	0.80 ± 0.03 ^a^
190	5.60 ± 0.19 ^a^	240	0.80 ± 0.02 ^a^

Significant differences (*p* < 0.05) are described with different alphabets (a,b).

**Table 4:** Molecular weights (kDa) of pyrolytically processed OMP-CND samples treated at temperatures ranging from 150 °C to 240 °C.

Under 200 °C		Above 200 °C	
Temperature (°C)	Molecular Weight (kDa)	Temperature (°C)	Molecular Weight (kDa)
150	602.20 ± 1.20 ^a^	200	623.20 ± 1.00 ^b^
160	599.20 ± 5.40 ^a^	210	618.40 ± 1.80 ^abcd^
170	611.80 ± 1.20 ^abcd^	220	608.50 ± 7.90 ^abcd^
180	614.60 ± 6.20 ^b^	230	540.10 ± 5.20 ^d^
190	620.60 ± 1.20 ^bc^	240	411.20 ± 6.50 ^d^

Significant differences (*p* < 0.05) are described with different alphabets (a,b,c,d).

**Table 5:** Antimicrobial effects of OMP-CND samples against *Escherichia coli* and *Staphylococcus epidermidis* based on bacterial growth.

Tested Samples	*Escherichia coli* (Million CFU/g)	*Staphylococcus epidermidis* (Million CFU/g)
Control	1075.0 ± 16.1 ^a^	13.5 ± 2.1 ^a^
1 *w*/*v*% of OMP-CND-FL-210	605.0 ± 7.1 ^b^	11.5 ± 0.7 ^a^

Significant differences (*p* < 0.05) are described with different alphabets (a,b).

## Data Availability

The original contributions presented in this study are included in the article. Further inquiries can be directed to the corresponding author.
